# Direct neuronal reprogramming of olfactory ensheathing cells for CNS repair

**DOI:** 10.1038/s41419-019-1887-4

**Published:** 2019-09-09

**Authors:** Xiu Sun, Zijian Tan, Xiao Huang, Xueyan Cheng, Yimin Yuan, Shangyao Qin, Dan Wang, Xin Hu, Yakun Gu, Wen-Jing Qian, Zhongfeng Wang, Cheng He, Zhida Su

**Affiliations:** 1 Institute of Neuroscience, Key Laboratory of Molecular Neurobiology of Ministry of Education and the Collaborative Innovation Center for Brain Science, Second Military Medical University, 200433 Shanghai, China; 2 Institutes of Brain Science and State Key Laboratory of Medical Neurobiology, Fudan University, 200032 Shanghai, China

**Keywords:** Spinal cord injury, Reprogramming

## Abstract

Direct conversion of readily available non-neural cells from patients into induced neurons holds great promise for neurological disease modeling and cell-based therapy. Olfactory ensheathing cells (OECs) is a unique population of glia in olfactory nervous system. Based on the regeneration-promoting properties and the relative clinical accessibility, OECs are attracting increasing attention from neuroscientists as potential therapeutic agents for use in neural repair. Here, we report that OECs can be directly, rapidly and efficiently reprogrammed into neuronal cells by the single transcription factor Neurogenin 2 (NGN2). These induced cells exhibit typical neuronal morphologies, express multiple neuron-specific markers, produce action potentials, and form functional synapses. Genome-wide RNA-sequencing analysis shows that the transcriptome profile of OECs is effectively reprogrammed towards that of neuronal lineage. Importantly, these OEC-derived induced neurons survive and mature after transplantation into adult mouse spinal cords. Taken together, our study provides a direct and efficient strategy to quickly obtain neuronal cells from adult OECs, suggestive of promising potential for personalized disease modeling and cell replacement-mediated therapeutic approaches to neurological disorders.

## Introduction

Due to the very limited regenerative ability in the adult central nervous system (CNS), the irreversible neuronal loss often leads to serious neural dysfunction after neurological disease or injury^1–3^. In spite of long-held dogmas on the impossibility of overcoming epigenetic barriers and changing the identity of differentiated cells, Takahashi and Yamanaka have successfully converted fibroblasts into induced pluripotent stem cells (iPSCs) in 2006^4^. Encouraged by the iPSCs, lineage conversion of differentiated somatic cells into functional neurons has recently attracted immense interest due to its possible application in neural regenerative medicine, disease modeling and drug identification. However, a major challenge for the potential applications of neuronal reprogramming in clinic is to identify an ideal cell source amenable to direct somatic cell-to-neuron conversion.

The accessibility and susceptibility are important criteria for identifying ideal starting cells for neuronal reprogramming. By genetic reprogramming, induced neuronal cells have been directly generated from different kinds of somatic cells, including hepatocytes^5^, pericytes^6^, fibroblasts^7–12^, and astrocytes^13–21^. Among them, fibroblasts and astrocytes have been extensively used for neuronal reprogramming experiments. For example, fibroblasts are morphologically heterogeneous mesenchymal cells that exist abundantly in connective tissues. They are easily accessible and have been successfully reprogrammed into various types of induced neurons^22,23^. As a distantly lineage-related somatic cell type, however, the reprogramming of fibroblast is a time-consuming process and suffers from low efficiency, which is regarded as limitations for clinical applications of fibroblast-induced neuronal cells. For cell transplantation/replacement therapy, of note, the non-reprogrammed fibroblasts may be detrimental to neural regeneration^24,25^. Astrocytes are the most abundant cell populations that exist throughout the central nervous system (CNS)^26^. Due to the proximity to neuronal lineage and capability to proliferate, astrocytes have been selected as starting cells to regenerate neurons in high efficiency. Nevertheless, they are difficulty to obtain from patients.

Olfactory ensheathing cells (OECs), a unique type of glial cells that derive from the olfactory placode, wrap olfactory axons, and support their continual regeneration from the olfactory epithelium to the bulb^27^. OECs exist in both the peripheral nervous system (PNS) and CNS, sharing many similar biological features and functions with astrocytes^28,29^. It have been well documented that OECs play key roles in the spontaneous growth of olfactory axons within the developing and adult olfactory nervous system^27^. Therefore, transplantation of OECs have emerged as promising strategy to promote neural regeneration after CNS injury^30^. Clinically, the olfactory mucosa represents a feasible source of autologous OECs. Based on these properties, we hypothesized that OECs may be an ideal starting cell type for neuronal reprogramming.

In the present study, we screened a series of transcription factors and found that Neurogenin 2 (NGN2) could rapidly and efficiently reprogram OECs into neuronal cells. These induced cells showed typical neuronal morphologies, neuron-specific marker expression, synapse formation, and electrophysiological properties. Their neuronal gene-expression profile was further confirmed by genome-wide RNA-sequencing analysis. The NGN2-mediated OEC-to-neuron conversion was a direct reprogramming process but not passing through a proliferative progenitor state. After transplantation into normal or injured spinal cord, these OEC-converted neurons could survive and become mature. Thus, we here identified OECs as an ideal cell type for direct and efficient reprogramming to obtain neurons that might be potentially used for cell-based therapy or disease modeling.

## Materials and methods

### Animals

Wild type C57/BL6J and the immunodeficient NOD SCID gamma (NSG) mice were obtained from Shanghai Ling Chang Biotech Co., Ltd. Animals were housed under a 12-h light/dark cycle and had ad libitum access to food and water. All animals in this study were handled in strict accordance with the guidelines recommended by the National Institutes of Health. The experimental procedures and protocols were approved by the Animal Experimentation Ethics Committee of the Second Military Medical University.

### Plasmids preparation and lentivirus production

Lentivirus was used to deliver the candidate factors. Candidate genes (SOX2, ASCL1, OLIG2, NEUROD1, BRN2, and NGN2) from human or mouse sources were amplified by PCR and sub-cloned into a third-generation lentiviral vector (pCSC-SP-PW-IRES/GFP) to generate pCSC-SP-PW-TF-IRES-GFP (TF: transcription factor; Supplementary Fig. 1), in which co-expressed GFP in the vector was used to visualize virus-infected cells. To specifically target OECs, the transcription factor NGN2 was also sub-cloned into a lentiviral vector in which gene expression was regulated by a human glial fibrillary acidic protein (hGFAP) promoter. The lentiviral vector and packaging plasmids (pMDL, VSV-G, and pREV) were transiently transfected into HEK293T cells to produce replication-deficient lentivirus. Lentivirus was collected and applied for cell reprogramming.

### Primary OEC culture and viral infection

Primary OECs were prepared from postnatal (P4), adult and aged C57BL/6 mice and purified by differential cell adhesiveness as previously described^31^. OECs isolated from olfactory bulb or olfactory mucosa was plated on uncoated 25-cm^2^ culture flasks two times, each for 36 h at 37 °C in 5% CO_2_. Three days later, the adhesive cells mainly composed of fibroblasts were discarded; the non-adhesive cell suspension was collected and then seeded onto poly-l-lysine-coated dishes. The OEC cultures were incubated with 15% fetal calf serum-containing DMEM/F-12 supplemented with 2 μM forskolin (Sigma) and 10 ng/mL bFGF (Sigma).

For neuronal reprogramming, OECs were seeded on gelatin/matrigel-coated culture vessels with or without glass coverslips. The following day, the cultured OECs were infected with the indicated lentivirus in the presence of 6 μg/mL polybrene. After 12–14 h, culture media were refreshed. Two days later, the virus-infected OECs were switched to neuronal induction medium (NM). The NM consists of DMEM:F12:Neurobasal (2:2:1), 0.8% N-2 (Invitrogen), and 0.4% B-27 (Invitrogen), in which forskolin (FSK, 10 μM) and dorsomorphin (DM, 1 μM) were supplemented. During the lineage reprogramming process, the induction medium was half-changed every other day.

### BrdU incorporation assay

BrdU incorporation assay was used to detect proliferating cells in culture. Cell cultures were incubated with 10 μM 5-bromo-2-deoxyuridine (BrdU, Sigma) for the indicated times. For BrdU staining, the cells were pretreated with 2 mol/L hydrochloric acid for 30 min at 37 °C to denature DNA. The excess hydrochloric acid was neutralized by incubation with 0.1 mol/L sodium tetraborate at room temperature for 10 min. By fluorescent staining with an anti-BrdU antibody, BrdU incorporation was detected in the cells.

### Immunofluorescence

For immunocytochemistry, cell cultures were fixed with 4% paraformaldehyde (PFA) in PBS for 20 min at room temperature. For immunohistochemistry, mice were anesthetized with 2% pentobarbital and then intracardially perfused with 4% PFA in PBS. Spinal cords were surgically dissected and post-fixed overnight at 4 °C. After cryoprotected with 30% sucrose at 4 °C for 48 h, spinal sections of spinal cords spanning the injection/injury sites were cut on a cryostat (Leica) set at 14 μm thickness. Fixed cells or spinal sections were permeabilized and blocked with 0.2% Triton X-100 and 3% BSA in 1 × PBS for 1 h, followed by incubation in the primary antibodies (Supplementary Table 1) overnight at 4 °C. The appropriate secondary antibodies conjugated to Alexa Fluor 488, 594, or 647 (Jackson ImmunoResearch) were used for indirect fluorescence. Nuclei were counterstained with Hoechst 33342 (Hst). Images were captured with a Nikon E600FN microscope or a Leica confocal microscopy.

### Quantitative real-time reverse-transcription polymerase chain reaction

For gene expression analysis, total RNA was extracted from cultured cells with Trizol reagent (Invitrogen) and the contaminating DNA was depleted with RNase-free DNase (Thermo Scientific Fermentas). The quantitative real-time reverse-transcription polymerase chain reaction (qRT-PCR) was performed using a MyiQ™ (Bio-Rad) with SYBR Green Realtime PCR Master Mix (TOYOBO Biotech). Using the 2^−ΔΔCt^ method, the gene expression was calculated and quantified after normalization to the expression of GAPDH. The qRT-PCR analysis was repeated at least three times.

### RNA sequencing and bioinformatics analysis

The RNA-seq assay was performed as previous protocol^32,33^. After OECs were infected with NGN2-expressing lentivirus, the induced neuronal cells were purified by FACS based on the co-expressed GFP in the vector. The total RNA was extracted from cultured OECs, OEC-derived induced neuronal cells and embryonic cortical neurons cells with Trizol reagent (Invitrogen). Sequencing was performed using the NGS (Next Generation Sequencing) technology with a Illumina Hiseq sequencer (Illumina Inc., San Diego, CA, USA). Based on GRCm38.p4 (mm10), genome mapping was carried out using Hisat2 (version: 2.0.4)^34^. The edgeR package^35^ was used to analyze the differentially expressed genes.

### Electrophysiology

Before electrophysiological analyses, OEC-converted neurons were co-cultured with astrocytes on glass coverslips for ~5–8 weeks. Based on the co-expressed GFP reporter, OEC-derived neurons were identified under epifluorescence. Whole-cell patch clamp recordings were carried out at 25 °C (room temperature) in a culture dish (CORNING) containing Ringers solution (135 mM NaCl, 3 mM KCl, 2 mM CaCl_2_, 1 mM MgCl_2_, 10 mM HEPES, and 11 mM Glucose at pH 7.4). Recording pipettes (approximately 3–5 MΩ) were filled with an intracellular solution (130 mM K-Gluconate, 15 mM KCl, 10 mM HEPES, 3 mM ATP-Mg, 0.3 mM GTP-Na, 0.2 mM EGTA, and 3 mM NaCl at pH 7.2). According to standard protocols, action potentials were recorded under current clamp in response to a series of current injections ranging from −200 to +200 pA with 50 pA increments and a 400-ms duration. Sodium currents were recorded under voltage clamp and elicited by a series of voltage steps ranging from −70 to +30 mV with 10 mV increments and a 200-ms duration. To block sodium current, a voltage-dependent Na^+^ channel blocker TTX (500 nM) was added into the culture dish and the voltage step was repeated to observe the TTX-sensitive currents. All the electrophysiological recordings were made using a patch amplifier (Multiclamp 700B) and data analysis was performed in a pClamp 10.2 software (Molecular Devices, Palo Alto, CA).

### Spinal cord injury model and cell transplantation

NSG mice at 2–3 months of age were used for the cell transplantation assay. OECs were infected with lentivirus expressing NGN2 and used for transplantation at 12 dpi. For normal spinal cord, animals were anesthetized with 2% pentobarbital (30 mg/kg) and a ~2-cm incision was made along the midline of the back. The dorsal surface of T_7–9_ spinal segment was exposed by a laminectomy. After surgery, OEC-converted neuronal cells (1 × 10^5^ per 1.5 μL) were stereotactically transplanted into the white matter (posterior funiculus) of spinal cord at each of the two locations 3-mm apart at T_8_ segment using a pulled glass micropipette with an inner diameter of 40 μm. For injured spinal cord, a crushed spinal cord injury (SCI) model was prepared as previously described^36^. Using a pair of forceps with a 0.45-mm spacer, the crush injury was induced by laterally compressing the T8 spinal cord for 15 s. Similarly, OEC-derived neuronal cells were manually injected into the spinal parenchyma at each of the two locations (1.5 mm proximal and distal to the lesion site) 2 weeks after SCI. The spinal cord-injured mice were subjected to manual bladder expression twice daily until recovery of reflexive bladder control. Spinal cords were collected at 7, 14 days or 1 month post injection for immunohistological analysis.

### Data and statistical analysis

Cells were quantified by counting twenty fields randomly selected from at least three separate experiments. At least 500 cells were counted. The quantitative data were expressed as mean ± SD. Statistical analysis was performed using Student’s *t*-tests. Differences were considered significant at *P* < 0.05.

## Results

### NGN2 converts OECs into neuronal cells

**Fig. 1:**
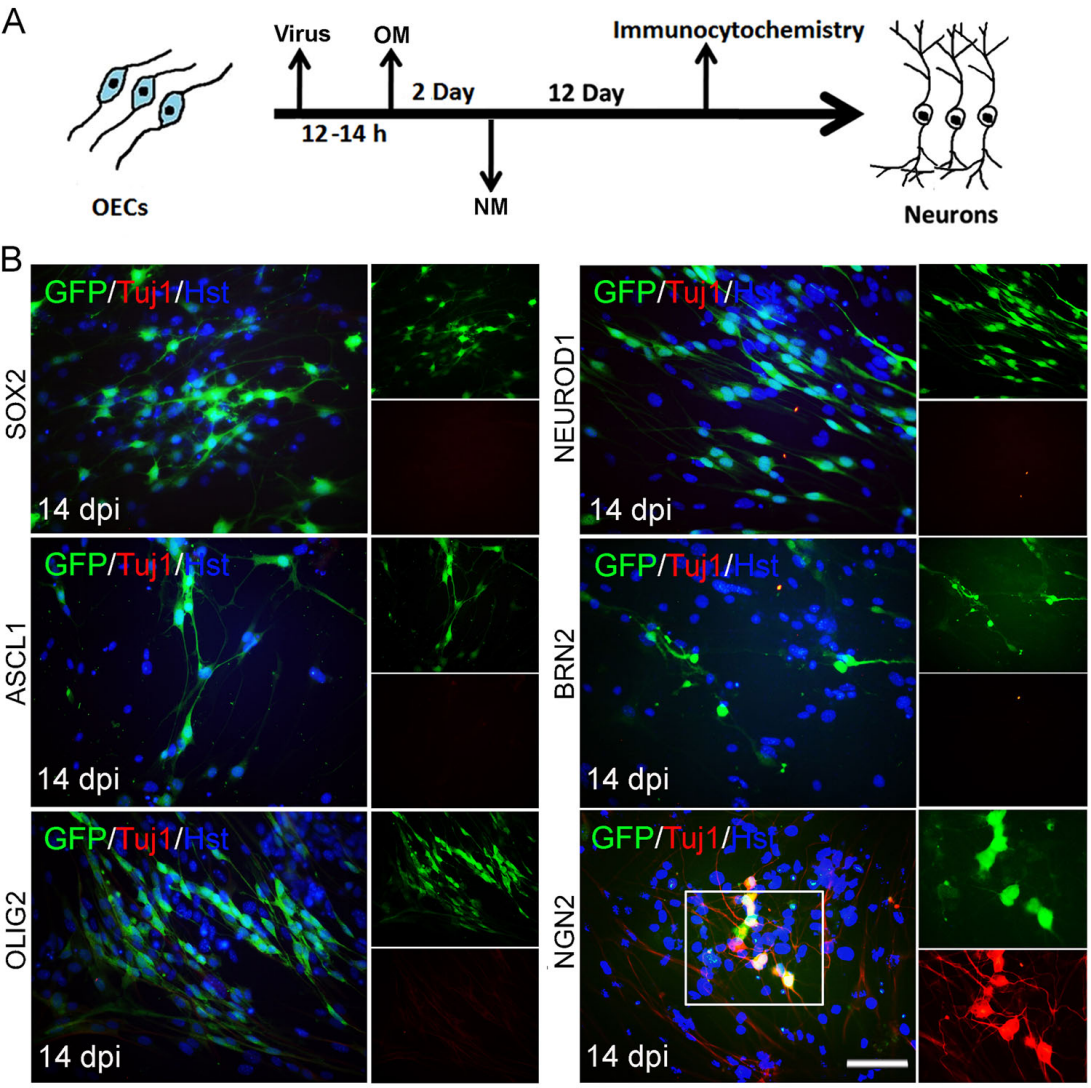
Induction of neuronal fate on OECs. **a** Experimental scheme of transcription factor-mediated neuronal reprogramming from OECs. OM, OEC culture medium; NM, neuronal induction medium. **b** Immunocytochemical analysis of induced neurons from OECs by ectopic expression of individual transcription factors, using antibody against Tuj1. Scale bar = 50 μm

Based on their roles in NSCs and/or neurogenesis, we selected and cloned 6 genes (SOX2, ASCL1, OLIG2, NEUROD1, BRN2, and NGN2) into lentiviral vectors for screening to convert OECs into induced neuronal cells (Fig. 1a). The OECs used for screening were isolated from the olfactory bulb of adult mouse. To exclude contamination of neuronal cells in primary OECs, the cultured cells were passaged at least once before being applied to reprogramming. Immunostaining showed that they uniformly expressed OEC markers P75, GFAP, and S100β but not neuronal markers including DCX, Tuj1, NeuN, vGlut-1, and GABA (Supplementary Fig. 2). Based on the expression of P75 or GFAP, the purity of OECs cultured in our experimental conditions was more than 95%. These cultured OECs were infected with lentivirus (CMV promoter) expressing the candidate factors. On the basis of the co-expressed green fluorescent protein (GFP), the infection efficiency was estimated to be about 83% (Supplementary Fig. 3), although it varied slightly between experiments. To analyze the ability to induce neuronal reprogramming, OEC-to-neuron conversion was initially examined by staining for the expression of pan-neuronal marker Tuj1. Interestingly, Fig. 1b showed that ectopic expression of NGN2 but none of the other 7 candidate genes trigger conversion of OECs into Tuj1-positive neuronal cells at 14 days post infection (dpi).

**Fig. 2:**
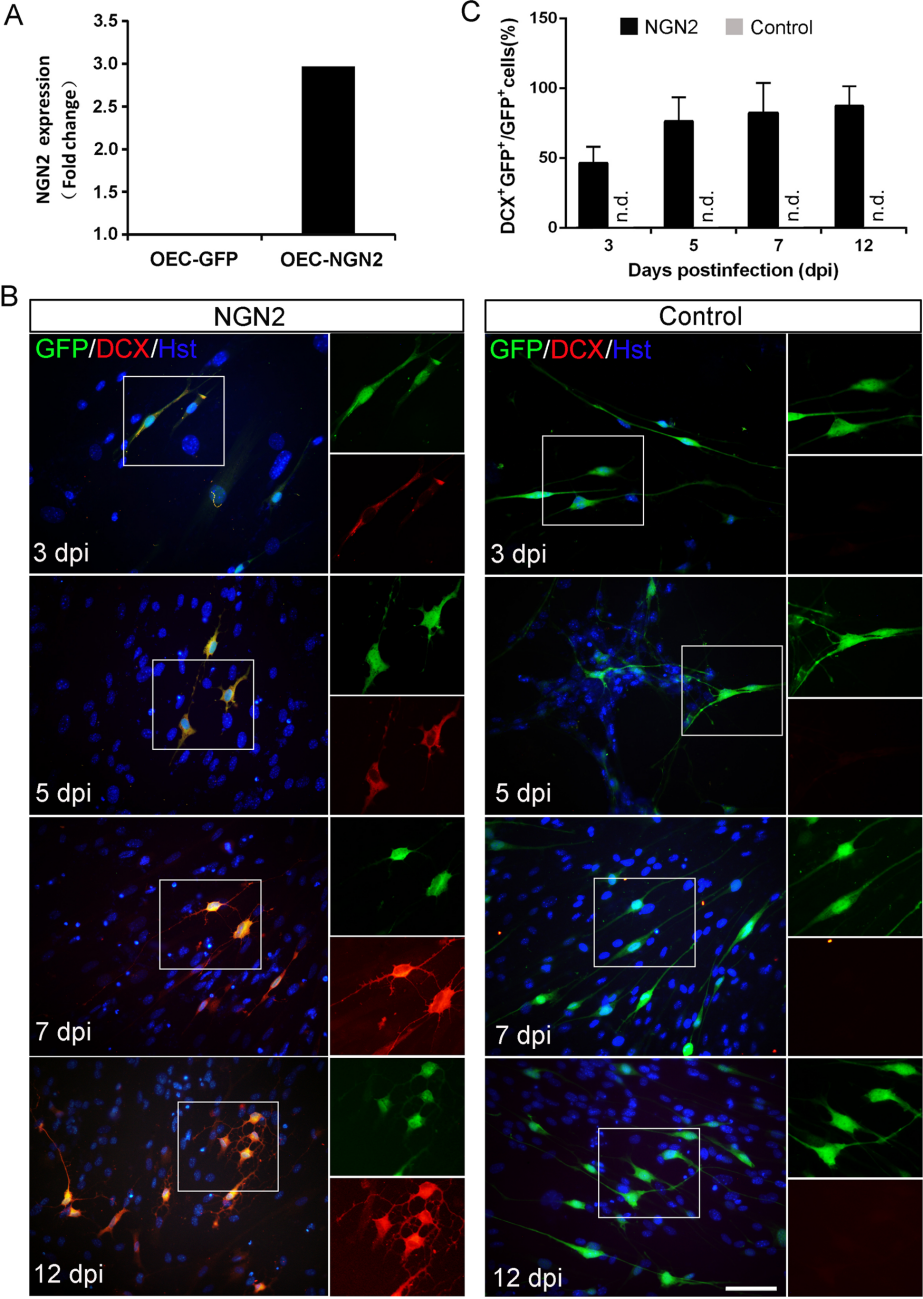
NGN2-induced lineage reprogramming of OECs into DCX^+^ cells. **a** RT-PCR confirming NGN2 expression in the virus-transduced OECs. **b** Representative micrographs of NGN2-induced neurons from OECs by staining with DCX at different time points. **c** Quantification of induced DCX^+^ cells during the indicated time course (*n* = 20 random fields from triplicate samples; n.d., not detected). Scale bar = 50 μm

**Fig. 3:**
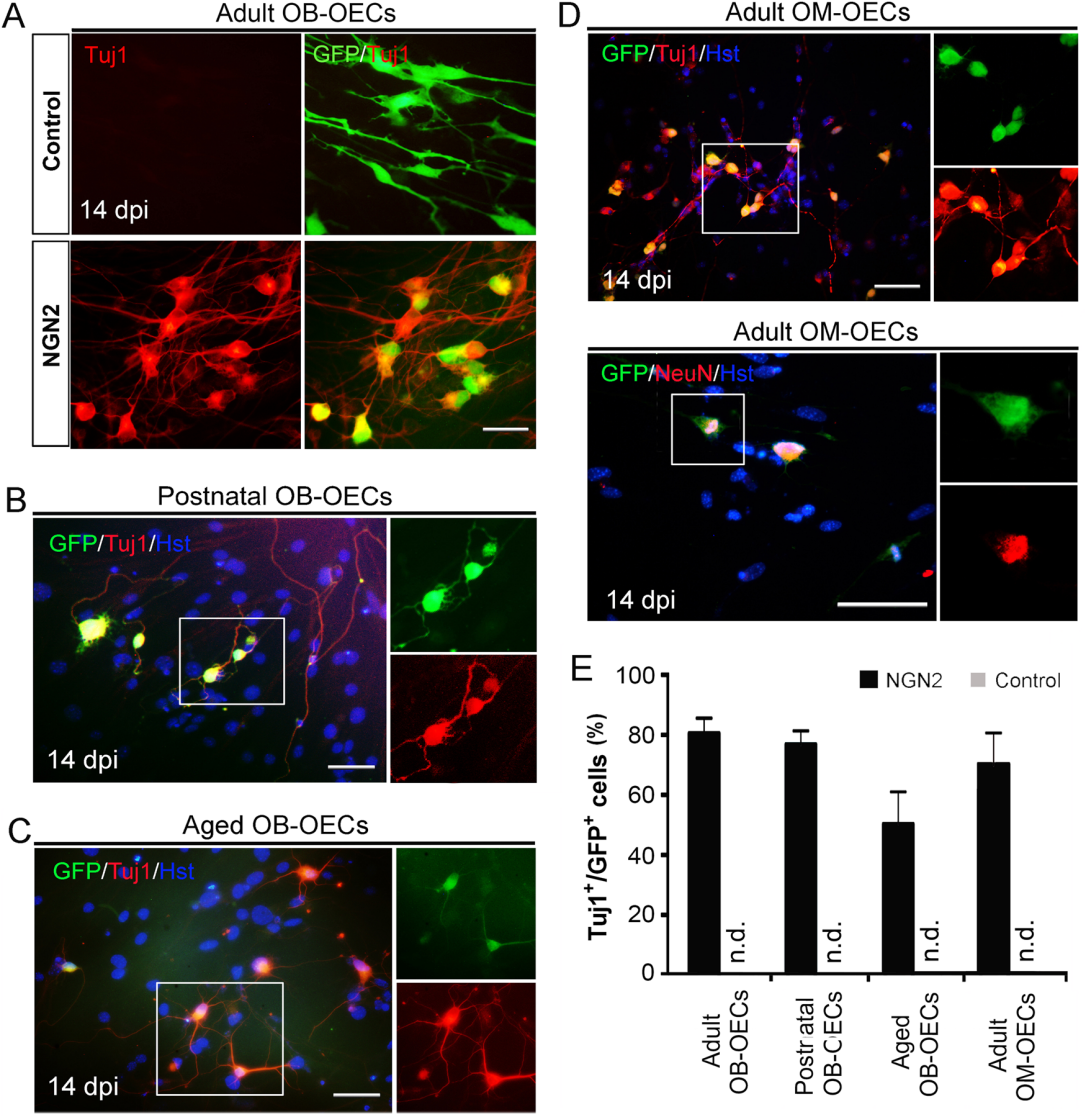
NGN2-induced neuronal reprogramming from OECs of different origins. **a–d** Immunocytochemical analysis of lineage reprogramming of OECs from adult olfactory bulb (Adult OB-OECs) (**a**), postnatal olfactory bulb (Postnatal OB-OECs) (**b**), aged olfactory bulb (Aged OB-OECs) (**c**), and adult olfactory mucosa (Adult OM-OECs) (**d**) by staining with antibodies against Tuj1 and NeuN at 14 dpi. **e** NGN2-induced neuronal reprogramming efficiency of OECs of different origins (*n* = 20 random fields from triplicate samples; n.d., not detected). Scale bar = 50 μm

We next further investigated the roles of NGN2 in inducing neuronal reprogramming of OECs. After infected with NGN2-expressing virus, RT-PCR analysis showed that the NGN2 gene was indeed ectopically expressed in cultured OECs (Fig. 2a). Of note, forced expression of NGN2-induced OECs to change morphology as early as 5 dpi (Supplementary Fig. 4A). They rapidly lost their flat (Astrocyte-like OECs) or fusiform (Schwann-cell-like OECs) morphology and adopt a neuronal morphology with long neurites. In sharp contrast, no significant change of cell morphology was observed in the control virus-infected group (Supplementary Fig. 4). Consistent with the morphological analysis, immnuocytochemistry revealed that some cells started to be positive for the immature neuronal marker doublecortin (DCX) at 3 dpi (Fig. 2b). The neuronal signal DCX gradually increased during the NGN2-mediated conversion process and showed a peak level at 12 dpi, whereas control virus treatment did not result in significant expression of DCX (Fig. 2b, c). At 14 dpi, immunostaining showed that about 81.1% NGN2-infected OECs were reprogrammed into Tuj1-positive neuronal cells (Fig. 3a, e), which is more efficient than NGN2-mediated astrocyte-to-neuron conversion (~60%)^14,20,21^. Importantly, to exclude the possibility that the NGN2-converted neuronal cells were from the contaminating fibroblasts in primary OECs, we infected the OEC cultures with a human GFAP (OEC marker) promoter-driven NGN2-expressing virus. Similarly, we found that NGN2-infected cells were also efficiently converted into induced neuronal cells (Supplementary Fig. 5). Together, these findings suggest that the transcription factor NGN2 alone is sufficient to rapidly and efficiently reprogram OECs into neuronal cells.

### Generation of neuronal cells from OECs of different sources

The olfactory system consists of olfactory bulb, olfactory pathway, and olfactory mucosa. In addition to the adult olfactory bulb-derived OECs (Adult OB-OECs), we also further evaluate if NGN2-induced neuronal cells can also be generated from postnatal olfactory bulb-derived OECs (Postnatal OB-OECs), aged olfactory bulb-derived OECs (Aged OB-OECs) and adult olfactory mucosa-OECs (Adult OM-OECs). Postnatal OB-OECs and aged OB-OECs were isolated from the olfactory bulb of 4-day-old mice and mice at >12 months of age, respectively (Supplementary Fig. 6A, B). Similar to the adult OB-OECs experiment, forced expression of NGN2-converted postnatal OB-OECs (77.1%) and aged OB-OECs (50.3%) into induced cells, displaying complex neuronal morphologies and expressing pan-neuronal marker Tuj1 (Fig. 3b, c, e). The olfactory mucosa represents an easily accessible source of autologous OECs. Therefore, we derived OM-OECs from the olfactory mucosa of adult mice (Supplementary Fig. 6C) and infected them with NGN2-expressing lentivirus. Interestingly, we found that the adult OM-OECs (71.3%) could be reprogrammed into Tuj1- positive induced cells, indicative of neuronal fate (Fig. 3d, e). These adult OM-OECs-derived neuronal cells were also immunoreactive for NeuN (Fig. 3d). All these results suggest that the NGN2-induced neuronal cells can be successfully generated from mouse OECs independent of their origin.

### Genome-wide transcriptional remodeling during lineage conversion

To further globally understand the reprogramming state, the genome-wide transcriptional profiling of OEC-converted induced neuronal cells was analyzed by RNA-sequencing.

**Fig. 4:**
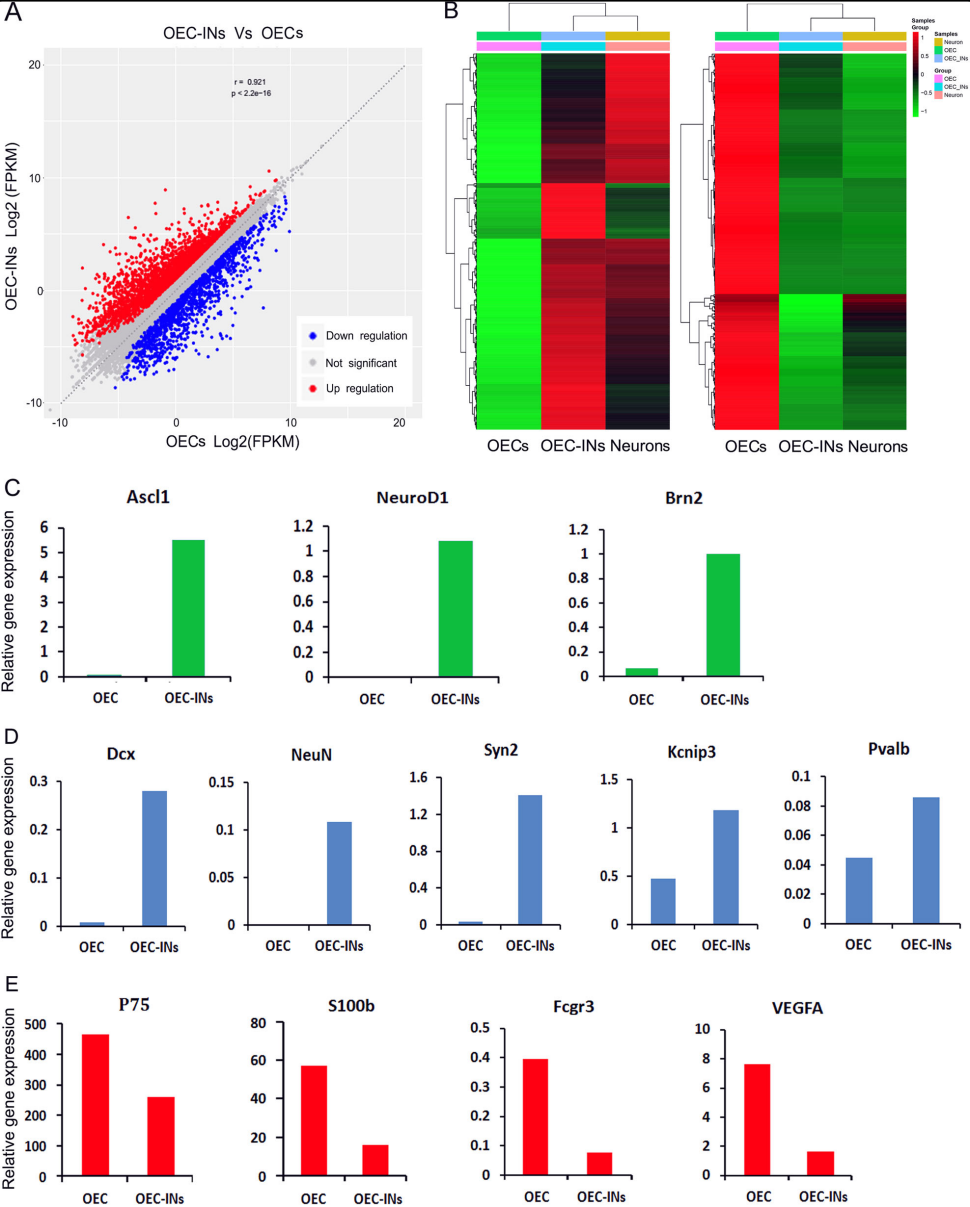
Transcriptome analysis of induced neuronal cells from OECs. **a** Scatterplots comparing gene expression levels between OEC-derived induced neurons (OEC-INs) and OECs (OECs). Up-regulation genes were highlighted in red; down regulation genes were highlighted in blue. **b** Heatmap of genes differentially expressed in RNA-sequencing analysis performed on OECs, OEC-INs, and cortical neurons (Neurons). Compared with OECs, the up-regulation genes (left panel) and down regulation genes (right panel) were identified in OEC-INs. **c**–**e** Gene expression (FPKM) of representative genes for neurogenic factors (**c**), neuronal lineage markers (**d**), and OEC markers (**e**), in OECs and OEC-INs at 14 dpi

At 14 dpi, OEC-derived induced neuronal cells (OEC-INs) were purified by fluorescence-activated cell sorting (FACS) and processed for RNA extraction and sequencing. Cortical neurons cultured from embryonic mouse (E18) served as positive control (Neurons; Supplementary Fig. 7). The transcriptome of OEC-INs was compared with those of OECs and cortical neurons. Scatter plots of the scores revealed a significant difference between OEC-INs and OECs, including 2604 up-regulated genes and 1416 down-regulated genes (Fig. 4a). By hierarchical cluster analysis, a similar gene expression pattern was observed between OEC-INs and cortical neurons (Fig. 4b). Although there were subtle differences in the global gene expression profiles of OEC-INs and cortical neurons, they were dramatically distinct from that of OECs. The heatmap of genes differentially expressed in RNA-sequencing analysis indicated that the transcription factor NGN2 induced a transcriptome shift from OECs toward neurons (Fig. 4b).

The generation of induced neuronal cells involves gradual silencing of starting cell-associated characteristic molecules and activation of neuron-associated characteristic molecules during the reprogramming process. As shown in Fig. 4c, ectopic expression of NGN2 resulted in a significant increase in the transcriptional levels of several neural transcription factors including Ascl1, NeuroD1, and Brn2.

Along with the activation of endogenous neural transcription factors, many genes with restricted neuronal expression were up-regulated in OEC-INs, such as DCX, NeuN, Syn2, Kcnip3, and Pvalb (Fig. 4d). In contrast, the OEC-related genes were generally down-regulated in OEC-INs (Fig. 4e). For example, the P75 and S100b transcriptional level was significantly reduced, coinciding with the activation of endogenous neural transcription factors and the up-regulation of neuronal genes. In conclusion, these findings suggested that forced expression of NGN2 induces neuronal cells by activating a multitude of neural transcription factors and neuronal genes while leading to a significant loss of OEC molecular hallmarkers.

### NGN2-mediated OEC-to-neuron conversion is a direct process

**Fig. 5:**
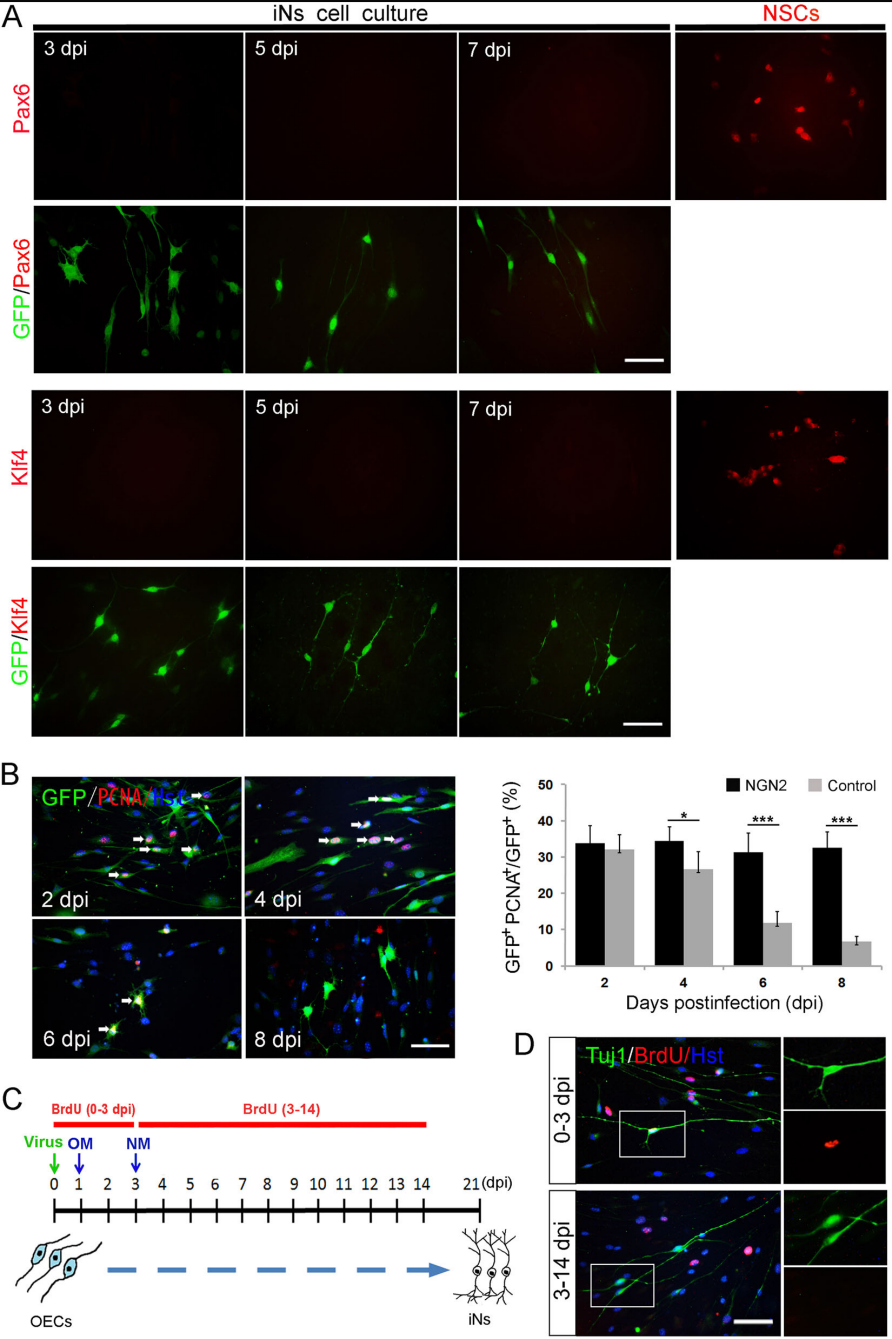
Immunocytochemical analysis of NGN2-mediated neuronal conversion process. **a** Lack of marker expression for neural progenitors during the reprogramming process. The expression in neural stem cells (NSCs) served as a positive control. **b** Time-course analysis of proliferating cells by staining with PCNA (*n* = 20 randomly selected fields from triplicate samples). **c**, **d** Incorporation of BrdU in NGN2-induced neuronal cells. Cells were treated with BrdU for the indicated duration after viral infection (**b**). OM, OEC culture medium; NM, neuronal induction medium. *P < 0.05, ***P < 0.001 by Student’s *t-*tests. Scale bar = 50 μm

To test whether the OEC-to-neuron conversion might pass through a neuroprogenitor intermediate, Pax6 and Klf4 signals were monitored during the NGN2-mediated reprogramming process. The time-course analysis of protein expression by immunocytochemistry showed that the neural progenitor markers Pax6 and Klf4 were not evident when examined at 3, 5, and 7 dpi, compared to NSCs (Fig. 5a). We next examined the cell proliferation during the lineage reprogramming. Cultured OECs were infected with NGN2-expressing lentivirus and stained with antibody against PCNA, a marker for proliferating cells, at 2, 4, 6, and 8 dpi (Fig. 5b). Among the NGN2-infected (GFP^+^) OECs, immunocytochemical analysis showed that PCNA-labeled proliferating cells decreased significantly after viral infection (Fig. 5b), suggesting that there was no expansion of progenitor cells during the OEC-to-neuron conversion. In addition, continuous BrdU labeling was also performed to further examine whether a proliferative progenitor state was involved in NGN2-mediated reprogramming. When BrdU was added into the culture medium at the first three days post virus infection, about 27% of OEC-derived neurons incorporated BrdU (Fig. 5c, d). In contrast, although non-transduced control cells were efficiently labeled by BrdU, few OEC-converted Tuj1^+^ cells were positive for BrdU after a long-term incorporation from 3-14 dpi (Fig. 5c, d), indicating that the OEC-to-neuron conversion resulted in cell cycle exit. Collectively, our results suggest that the NGN2-induced reprogramming directly converts OECs into neuronal cells.

### Functional maturation of OEC-converted neuronal cells

**Fig. 6:**
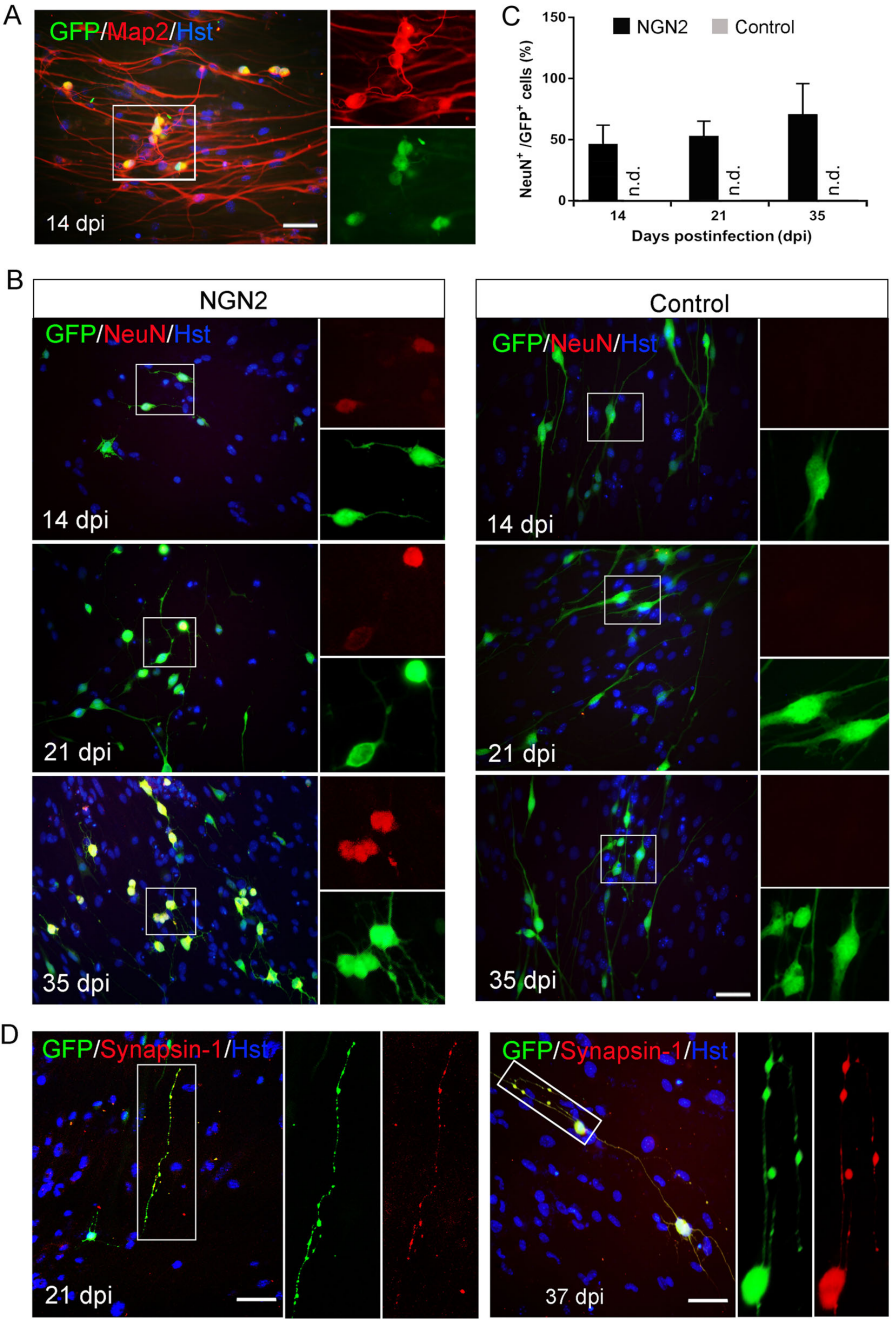
Maturation of NGN2-induced neurons from OECs. **a** Representative micrographs of mature NGN2-induced neurons from adult OECs by staining with mature neuronal marker Map2 at 14 dpi. **b**, **c** Time-course analysis of the maturation of induced neuronal cells by staining with mature neuronal marker NeuN (*n* = 20 randomly selected fields from triplicate samples). **d** Expression of the presynaptic marker synapsin-1 in the converted cells at 21 or 37 dpi. Scale bar = 50 μm

We next asked whether the OEC-converted neuronal cells would become functionally mature. Morphologically, time-course observation revealed that forced expression of NGN2 in OECs induced dramatic changes in their appearance, ultimately acquiring a mature neuron shapes with multiple and complex processes (Supplementary Fig. 4). As pan-neuronal makers, DCX is restricted to neuroblasts and immature neurons and Tuj1 is broadly expressed in both immature and mature neurons. Although OEC-converted neuronal cells were confirmed to express these two markers, it does not indicate that they will become mature. Therefore, we further examined neuronal maturation by immunostaining with mature neuron-specific markers, Map2 and NeuN. These two markers were undetected in both primary OECs (Supplementary Fig. 2C, D) and control virus-infected OECs (Fig. 6b, c). Immunocytochemistry showed that NGN2-induced cells robustly expressed the mature neuronal marker Map2 as early as 14 dpi (Fig. 6a). The expression of NeuN was measured at 14, 21, and 35 days after induction. As shown in Fig. 6b, c, NeuN-positive mature neurons were indentified in NGN2-infected cells (GFP^+^) and their number gradually increased over time, around 48%, 60%, and 75% at 14, 21, and 35 dpi, respectively. Of note, presynaptic neuronal marker synapsin-1 (SYN1) was detected in discrete puncta in OEC-converted neuronal cells, suggesting the establishment of synaptic termini (Fig. 6d).

**Fig. 7:**
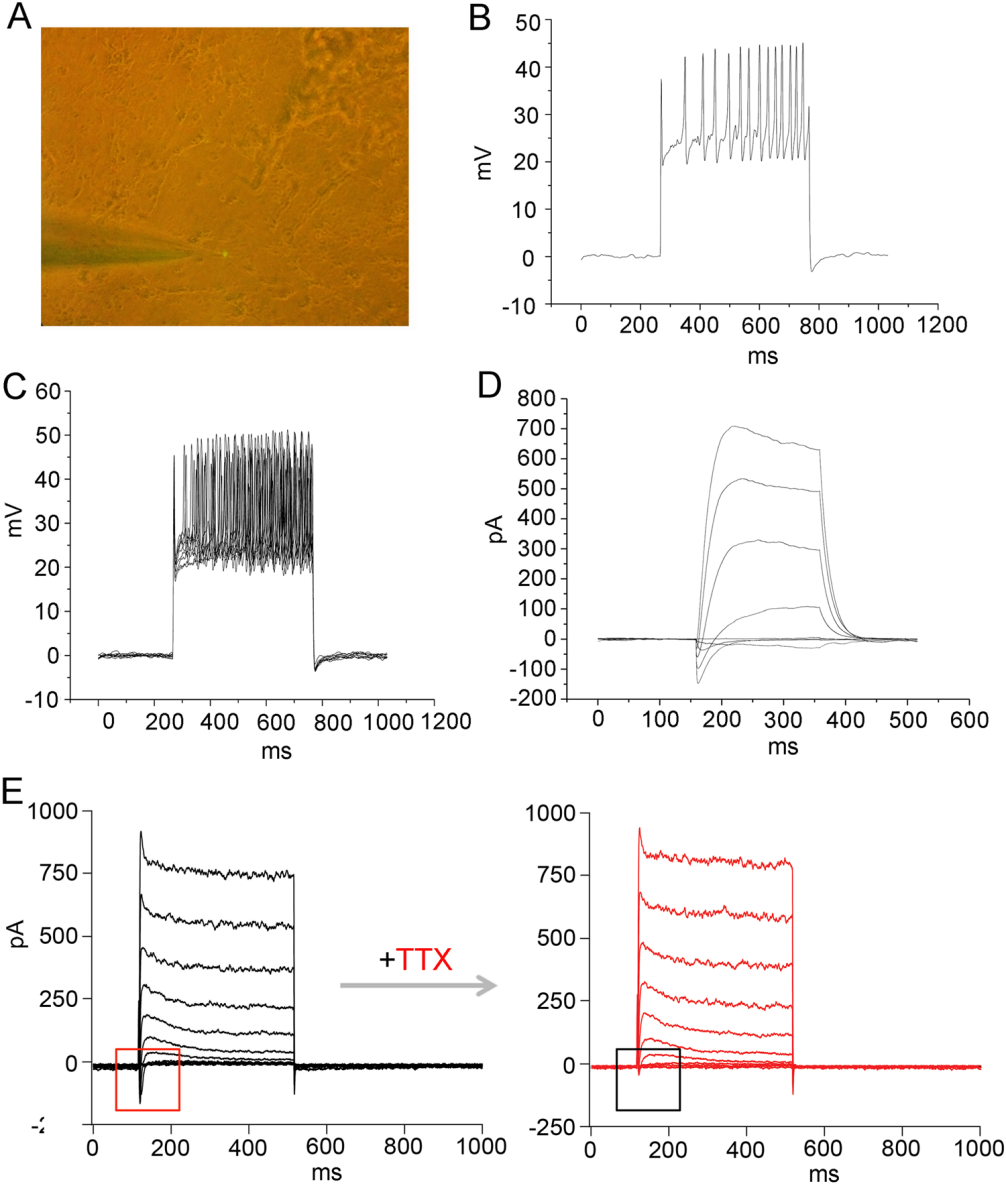
Electrophysiological properties of NGN2-induced neurons from OECs. **a** A representative visual field of the patched neuron containing GFP fluorescence. **b** Single action potential (AP) firing of an induced neuron which was co-cultured with astrocytes for 34 days in response to injection of a suprathreshold current pulse. **c** Multiple AP firing elicited by repetitious depolarizing current stimuli. **d** Voltage-clamp traces showing fast inward current and persistent outward current on depolarization. **e** Tetrodotoxin (TTX)-sensitive sodium currents. The voltage-gated Na^+^ currents recorded from an induced neuron (left panel) and subsequently blocked by TTX (right panel)

In addition to morphological properties and mark expression, characteristic electrophysiology is also the defining feature of mature neuronal identity. When co-cultured with astrocytes for about 30 days, the NGN2-induced cells with complex neuron morphology displayed characteristic electrophysiological properties for functionally mature neurons (Fig. 7). Single and multiple action potentials could be elicited in response to injection of a suprathreshold current pulse and repetitious depolarizing current stimuli, respectively (Fig. 7b, c). Moreover, fast inward current and persistent outward current were also induced by depolarizing voltage steps (Fig. 7d). Importantly, the inward currents could be blocked by the sodium channel-sensitive blocker tetrodotoxin (TTX), suggestive of their sodium current identity (Fig. 7e).

**Fig. 8:**
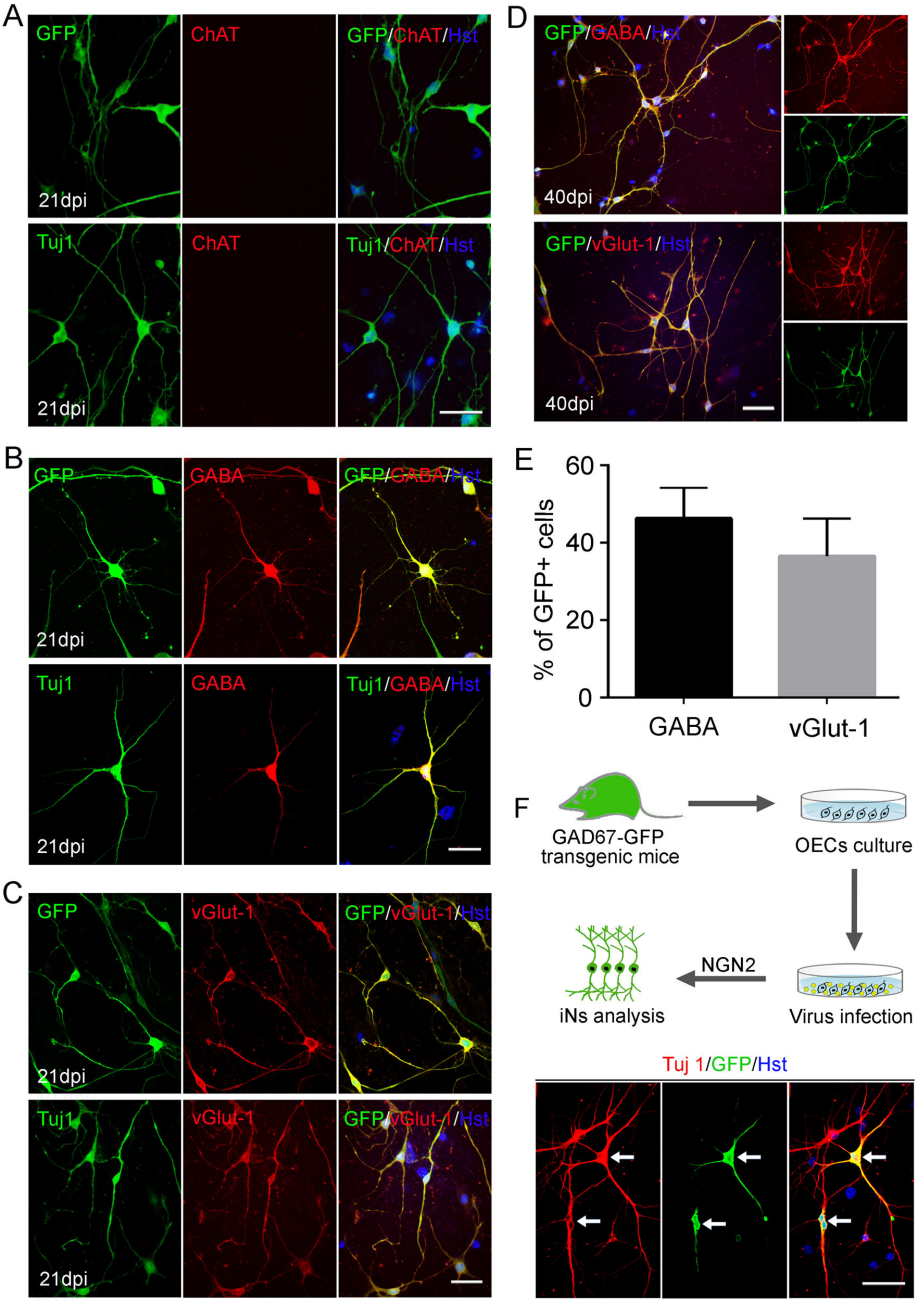
Subtype analysis of NGN2-induced neurons from OECs. **a** No expression of ChAT was detected in the induced neurons at 21 dpi. **b**, **c** Ectopic NGN2-induced OECs reprogramming into GABA^+^ (**b**) or vGlut1^+^ (**c**) neurons at 21 dpi. **d** Representative images of NGN2-induced GABA^+^ or vGlut1^+^ neurons at 40 dpi. **e** Quantification of the percentage of GFP^+^ cells expressing GABA or vGlut1 over the total of GFP^+^ cells (*n* = 20 randomly selected fields from triplicate samples). **f** Induction of GABAergic neurons from OECs of GAD67-GFP transgenic mice. OECs were cultured from olfactory bulb of adult GAD67-GFP transgenic mice, and then infected with NGN2-expressing lentivirus (without co-expressing GFP) to induce neuronal reprogramming. Immunocytochemical analysis was performed at 21 dpi. Scale bar = 50 μm

The cellular identity of OEC-coverted neurons was analyzed by immunostaining with neuronal subtype markers. At 21 dpi, no expression of choline acetyltransferase (ChAT), a marker for cholinergic motor, was detected in the induced neurons (indicated by the expression GFP and Tuj1) (Fig. 8a). However, confocal analysis showed that they were positive for GABA (inhibitory neuron marker) or vGlut-1, (excitatory neuron marker) (Fig. 8b–d). Quantitatively, the GABA^+^ and vGlut-1^+^ neurons accounted for 46 and 36%, respectively (Fig. 8e). The GABAergic neuronal identity was further demonstrated by gene-reporter mice (Supplementary Fig. 8A). OECs were cultured from the olfactory bulb of adult GAD67-GFP transgenic mice. Immunocytochemistry confirmed that there was no GFP-labeled cell and neither Tuj1^+^ nor GABA^+^ neurons were detected in these cultures (Supplementary Fig. 8B, C). These cultured OECs were infected with a NGN2-expressing lentivirus without co-expressing GFP. After forced expression of NGN2 in these OECs, a fraction of them were converted into GFP-positive neurons, suggestive of GABAergic neuronal identity (Fig. 8f). Taken together, these data indicate that ectopic expression of NGN2 in OECs converts them into a mixed population of functionally mature neurons.

### Transplantation of OEC-converted neuronal cells into mouse spinal cord

**Fig. 9:**
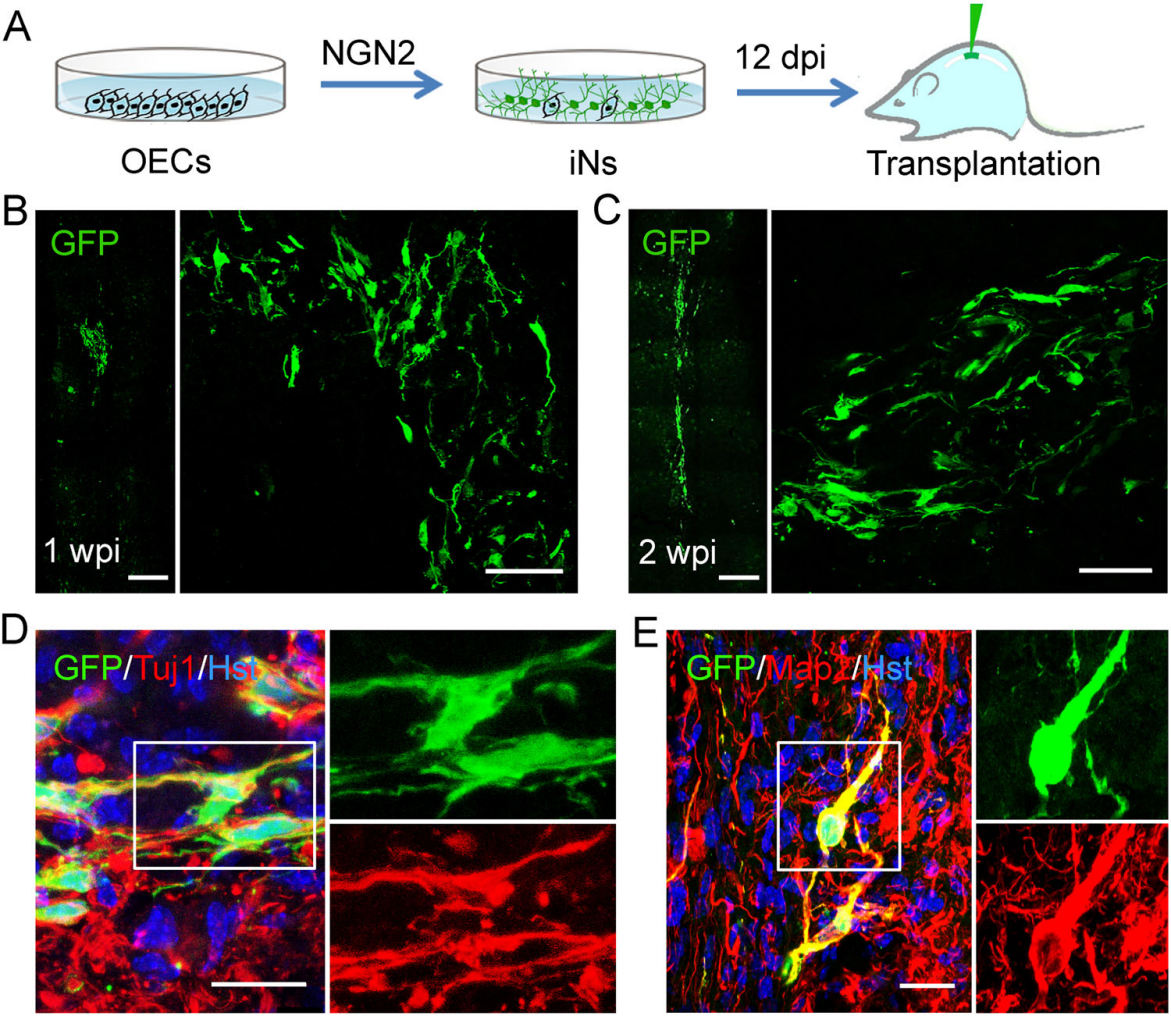
Transplantation of OEC-converted neurons into normal adult spinal cord. **a** Schematic diagram showing the experimental procedure of intraspinal injection of induced cells. **b**, **c** Survival of GFP-labeled induced cells at 1 and 2 weeks post injection (wpi). **d**, **e** Immunohistochemical analysis of OEC-derived neurons by staining with antibodies against Tuj1 and Map2 in the spinal cord of NOD-SCID mice. Scale bar, 100 μm for **b**, **c**; 50 μm for **d**, **e**

Cell transplantation assays were performed to further investigated whether OEC-converted neurons could survive and mature in vivo. At 12 days after initial infection of OECs with NGN2-expressing lentivirus, we harvested these cells and injected them into the normal spinal cord of adult mouse (Fig. 9a). The co-expressed GFP in virus-infected cells was used to trace OEC-derived neurons and distinguish them from the pre-existing mouse spinal neurons. One week after cell transplantation, immunohistochemical analysis of longitudinal sections showed a cluster of GFP-labeled cells were observed around the injection site (Fig. 9b), suggesting that the NGN2-induced cells successfully survived in the mouse spinal cord. These GFP-labeled injected cells could be still detected in vivo at 2 weeks after cell injection (Fig. 9c). Of note, they had migrated out of the injection site and distributed in a broad area, especially in the rostral and caudal direction (Fig. 9c). Importantly, Fig. 9d, e showed that a fraction of the GFP-labeled induced cells were immunopositive for neuronal markers Tuj1 and Map2, suggesting that they might have been converted into mature neurons after transplantation into the normal spinal cord.

**Fig. 10:**
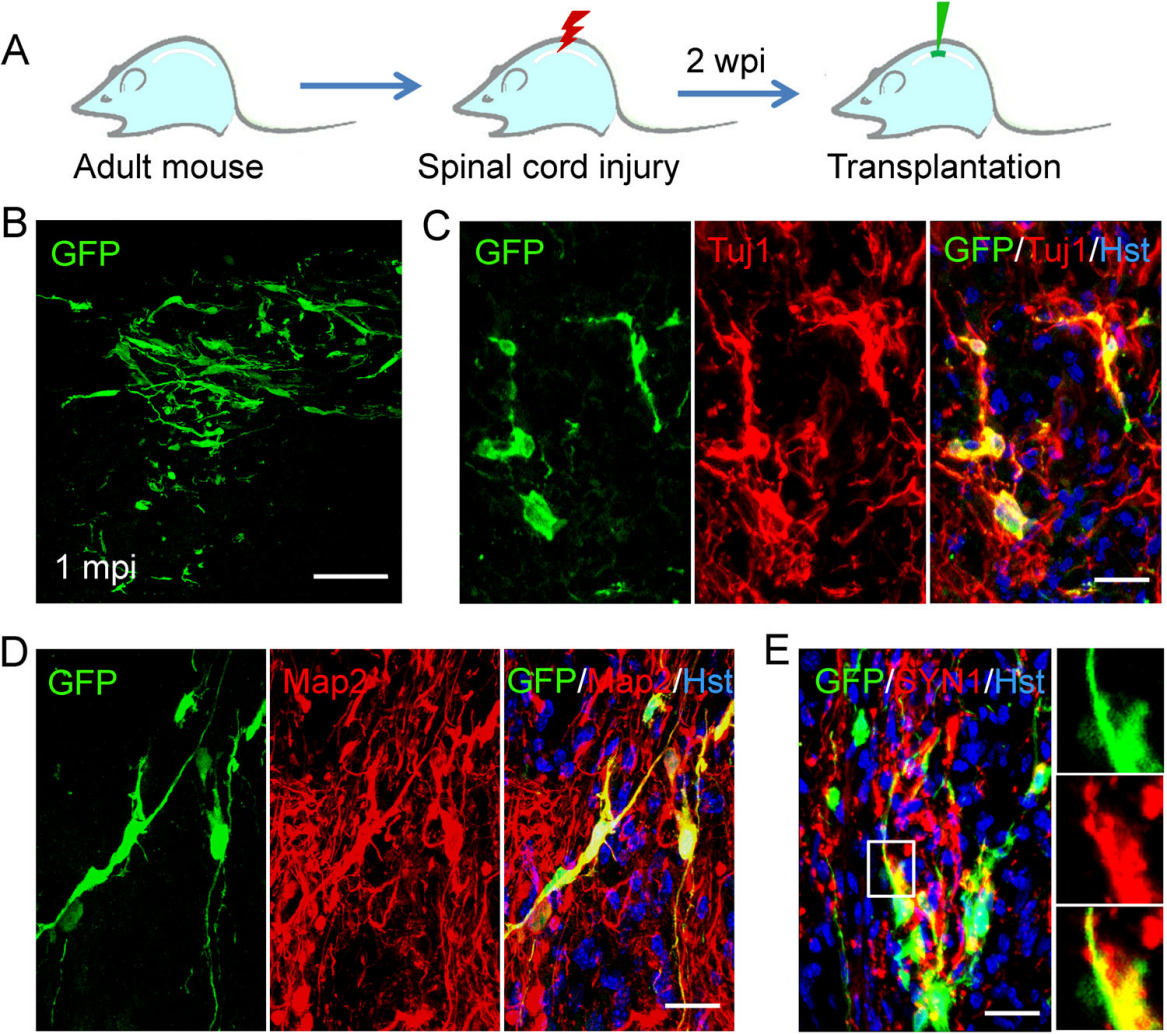
Transplantation of OEC-converted neurons into injured adult spinal cord. **a** Schematic diagram showing the experimental procedure of stereotactical injection of induced cells into crushed spinal cord. **b** Survival of GFP-labeled induced cells at one month post injection (mpi). **c**–**e** Immunohistochemical analysis of OEC-derived neurons by staining with antibodies against Tuj1, Map2, and synapsin-1 (SYN1) in the spinal cord of NOD-SCID mice. Scale bar, 100 μm for **b**; 50 μm for **c**–**e**

SCI results in the formation of a complex microenvironment around the injury site, including massive cell death, inflammation, and reactive gliosis, which is drastically different from that of normal spinal cord. To determined whether OEC-derived neurons can survive and mature in the pathological microenvironment, we also injected them into the damaged spinal cord. A clinically relevant SCI model was established by crushing the spinal cord at the T_8_ level and the transplantation of NGN2-induced cells was performed 2 weeks post injury (Fig. 10a). One month later, these implanted cells (GFP^+^) were found to survive in the injured spinal cord (Fig. 10b); and they expressed Tuj1 and Map2, suggestive of mature neuronal identity (Fig. 10c, d). Importantly, some dense bouton-like terminals co-stained with SYN1 and GFP were observed among the survived OEC-converted neurons (Fig. 10e), indicating the formation of synapses. Together, these in vivo experiments suggest that the OEC-derived neurons can survive and mature in the normal or injured spinal cord of adult mouse.

## Discussion

Recently, neuronal replacement using stem-cell-derived or lineage-reprogramming-derived cell products have raised the possibility of repairing the damaged or diseased CNS^37^. Although induced neuronal cells can be directly reprogrammed from distantly related somatic cells, as distant as cell types representing different germ layers^38–40^, it is still a challenge for identifying an ideal donor cell type amenable to neuronal reprogramming. As an alternative to fibroblasts and astrocytes, we here reported that OECs could be rapidly and efficiently converted into neurons by direct lineage reprogramming with the single transcription factor NGN2.

OECs, a unique type of macroglia in the olfactory system, share many morphological, molecular, and functional properties with astrocytes. They are easily accessible from the patient’s olfactory mucosa. Due to the strong proliferative ability, OECs can be expanded quickly in culture. Therefore, they are considered as a preferred source for neuronal reprogramming. In present study, indeed, we provided evidence that OECs could be successfully reprogrammed into neuronal cells by NGN2 with a high efficiency (about 80%). Of note, this OEC-to-neuron conversion was independent of the donor cell source and animal age. In addition, it has well been demonstrated that OECs can produce a range of molecules associated with extracellular matrix (ECM) and trophic factors, suggestive of a prime therapeutic candidate for use in neural repair^27^. Genetic engineering of OECs with the over expression of NGN2 remarkably increased the secretion of trophic factors, including GDNF, BDNF, and NGF, and showed substantial neuroprotective effects against damaged neurons^41^. Therefore, although forced expression of NGN2 did not convert all OECs into neurons, the non-reprogrammed OECs might be beneficial for the survival of the reprogrammed neurons. For cell-based transplantation, the non-reprogrammed OECs together with the reprogrammed neurons might also synergistically contribute to neural repair.

NGN2, a transcription factor that belongs to the basic helix-loop-helix (bHLH) family, is highly expressed in the developing CNS and regulates commitment of neural progenitors to a neuronal fate^42,43^. Ladewig et al. reported that ectopic expression NGN2 and ASCL1 (achaete-scute complex-like 1) in human postnatal fibroblast could reprogram them into functional neuron-like cells, which were enhanced by combining with small molecule–based inhibition of glycogen synthase kinase-3b and SMAD signaling^44^. Liu and colleagues showed that NGN2 combined with two small molecules (forskolin and dorsomorphin) could efficiently convert human fetal lung fibroblasts into cholinergic neurons^45^. In addition, forced expression of NGN2 directed postnatal cortical astrocytes to generate synapse-forming glutamatergic neurons^14^. In our study, pro-neuronal gene NGN2 was shown to convert OECs into a mixture of glutamatergic and GABAergic neurons. This result further highlights the significance of extrinsic (induction factors) and intrinsic (cellular context) cues on neuronal fate determination during the reprogramming process, although the underlying mechanisms remain unknown. Interestingly, our study revealed forced expression of NGN2 in OECs resulted in a significant increase of the mRNA expression of pro-neural genes including ASCL1, NEUROD1, and BRN2. These up-regulated pro-neural genes might synergize with NGN2 to induce neuronal fate on OECs.

Compared with iPSC approach, direct lineage reprogramming can avoid much of the carcinogenic risks when the induced cells are transplanted in vivo. In our study, the NGN2-induced OEC-to-neuron conversion was a rapid process. After forced expression of NGN2, the OECs rapidly morphed into neuron-like cells. The DCX-positive immature neurons were induced as early as 3 dpi and it was only 2 weeks for them to become Map2^+^ and NeuN^+^ mature neurons. This rapid conversion hinted that it might be a direct lineage reprogramming. In fact, our time-course analysis of the multipotent stem cell markers and cell proliferation confirmed that NGN2 directly reprogrammed OECs into neurons without passing through a progenitor intermediate. Furthermore, our study also showed that the OEC-converted neurons were functional. The RNA-seq data revealed that the NGN2-induced neuronal cells shared a highly similar gene expression pattern to cultured primary cortical neurons. Immunocytochemically, these induced cells were stained positive for mature neuronal markers Map2, NeuN, and synapsin-1. Electrophysiologically, the OEC-derived neuronal cells were shown to possess the functional properties, such as the ability to fire action potentials and the induction of membrane current. Importantly, graftings in adult mouse indicated that the OEC-converted neurons could survive in the normal and even injured spinal cord where they were able to migrate out of the transplanted area. Although a detailed functional analysis is needed for the implanted neurons, the detection of punctate synapsin-1 expression was indicative of their ability to form synaptic connections in vivo.

In summary, a key finding of the present study is that OECs, an easily accessible source, represent an ideal target for neuronal reprogramming. They can be rapidly, efficiently and directly converted into functional neurons, shedding light on their potential use for personalized disease modeling and cell replacement-mediated therapeutic approaches to neurological disorders.

## Supplementary information

Supplementary Material
