# Supplementary material for: Direct neuronal reprogramming of olfactory ensheathing cells for CNS repair
Source: Cell Death Dis. 2019 Sep 9;10(9):646. doi: 10.1038/s41419-019-1887-4 (PMC6733847; doi:10.1038/s41419-019-1887-4)
Supplement: Supplementary file 1 — Supplementary Material [file 41419_2019_1887_MOESM1_ESM.doc]

**Supplementary Material**

**Direct Neuronal Reprogramming of Olfactory Ensheathing Cells for CNS Repair**

# Xiu Sun, Zijian Tan, Xiao Huang, Xueyan Cheng, Yimin Yuan, Shangyao Qin, Dan Wang, Xin Hu, Yakun Gu, Wen-Jing Qian, Zhongfeng Wang, Cheng He, Zhida Su

**
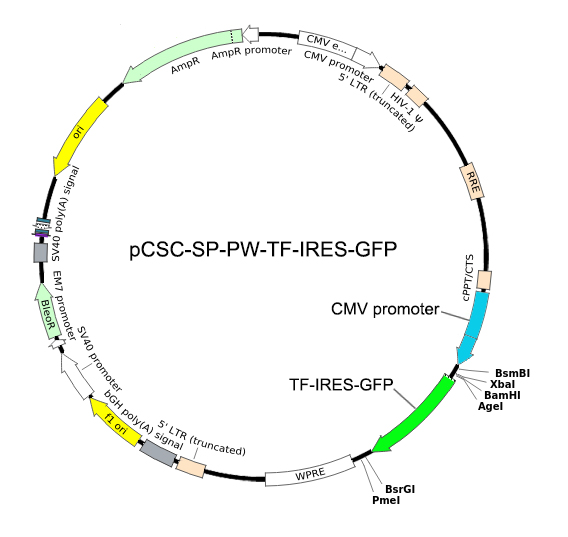
 Supplementary Figure 1 Plasmid construction.** Transcription factor (TF) was sub-cloned into a third-generation lentiviral vector (pCSC-SP-PW-IRES/GFP) to generate pCSC-SP-PW-TF-IRES-GFP.


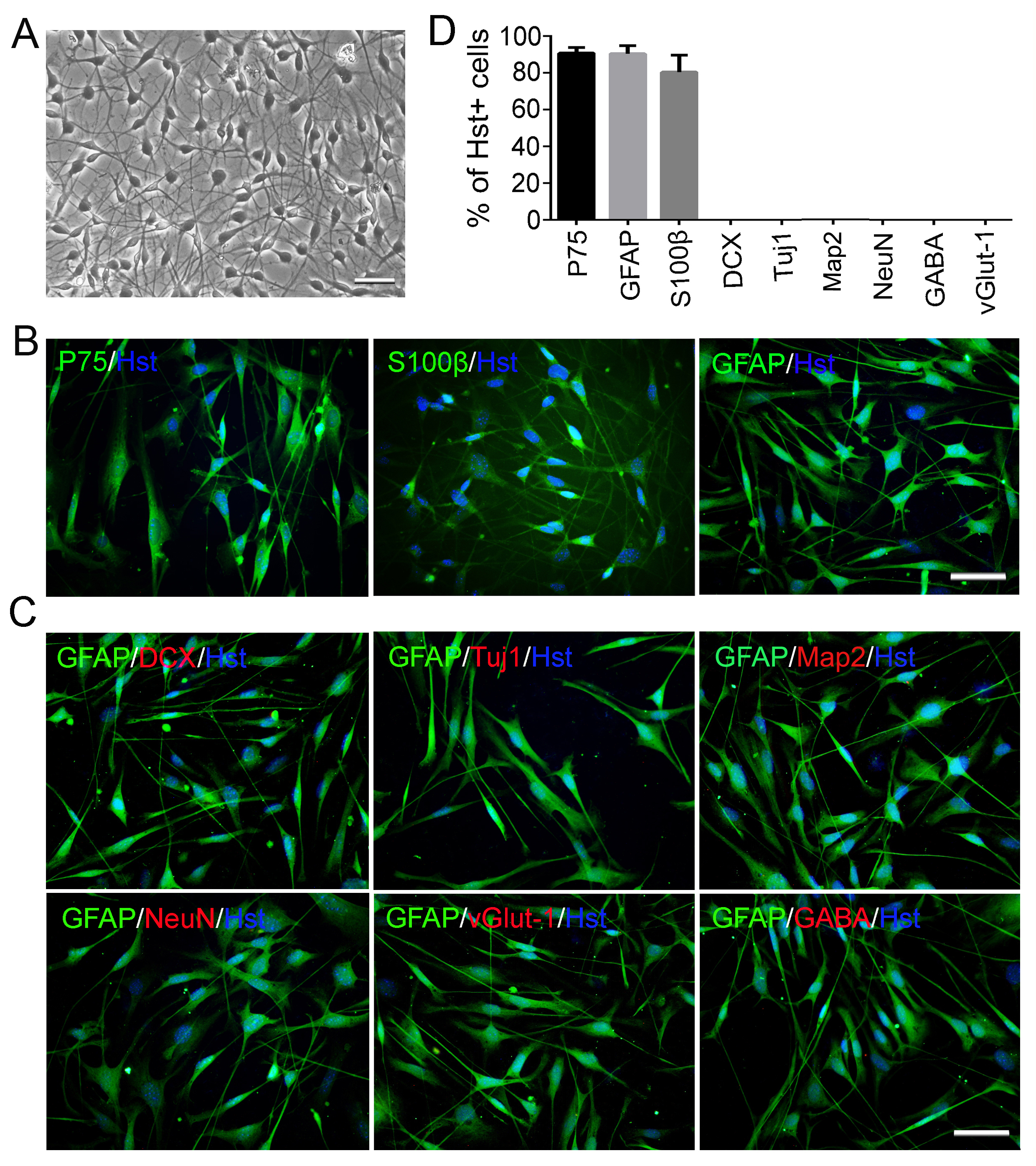


**Supplementary Figure 2 OECs cultured from olfactory bulb of adult mouse. (A)** Light microphotograph of adult mouse OECs cultured for 1 week. **(B)** The purity of cultured adult mouse OECs was determined by staining with antibodies against P75, GFAP and S100β. Nuclei were counterstained with Hoechst 33342 (Hst). **(C)**The cultured OECs did not express neuronal marks including DCX, Tuj1, Map2, NeuN, GABA and vGlut-1. **(D)** Quantification of the percentage of P75^+^, GFAP^+^, S100β^+^, DCX^+^, Tuj1^+^, Map2^+^, NeuN^+^, GABA^+^ and vGlut-1^+^ over Hst^+^ cells (n = 20 randomly selected fields from triplicate samples). Scale bar, 100 μm for (A); 50 μm for (B, C).


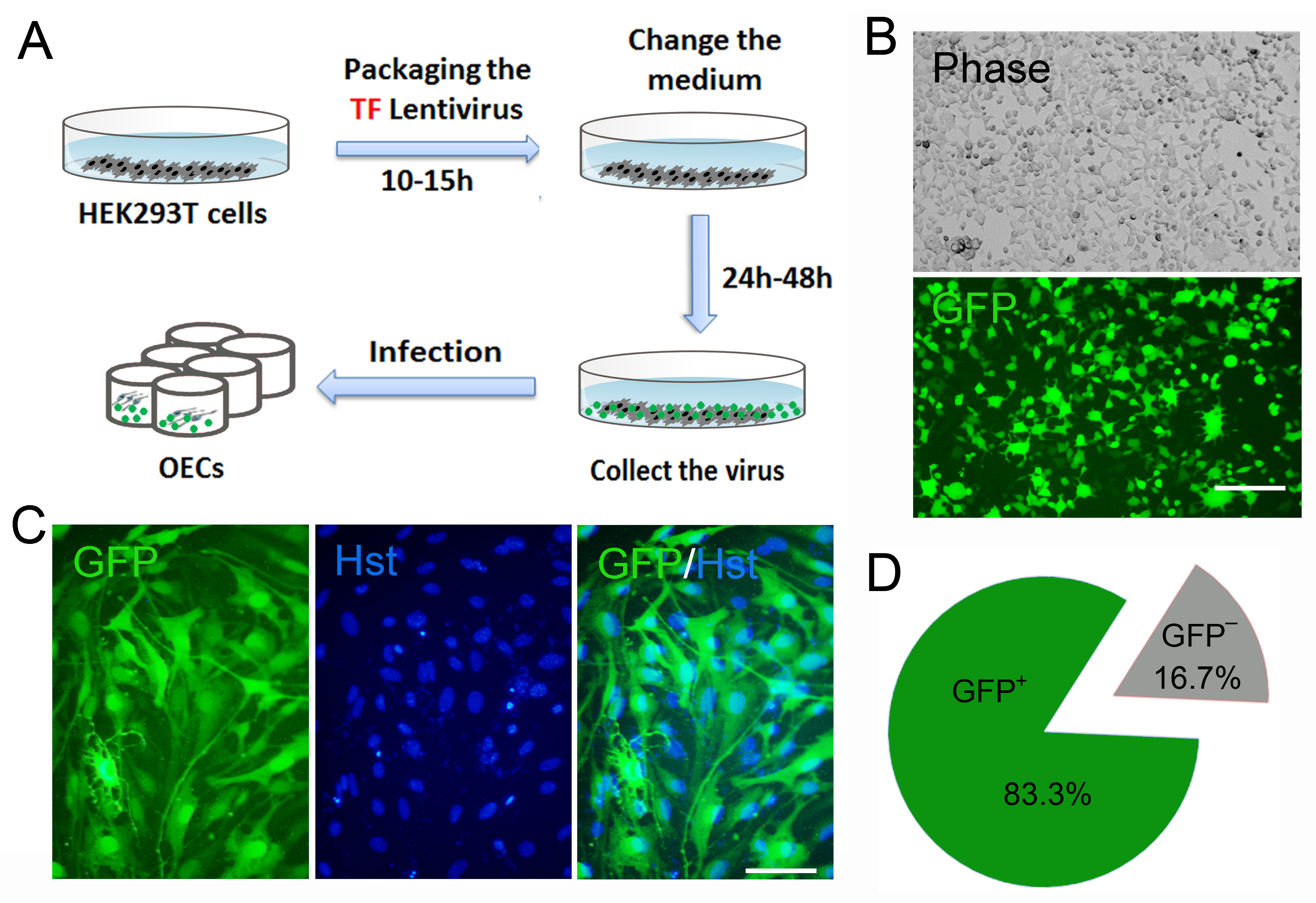


**Supplementary Figure 3 Lentivirus preparation and infection. (A)** Experimental scheme of lentivirus packaging, collection and infection. TF, transcription factor. **(B)** HEK293T cells (human embryonic kidney cells) were used to package the TF-expressing lentivirus. (C, D) Infection of OECs with lentivirus. Forty-eight hours later, the lentivirus-mediated infection efficiency was determined by measuring the coexpressed GFP. Scale bar, 100 μm for (B); 50 μm for (C).


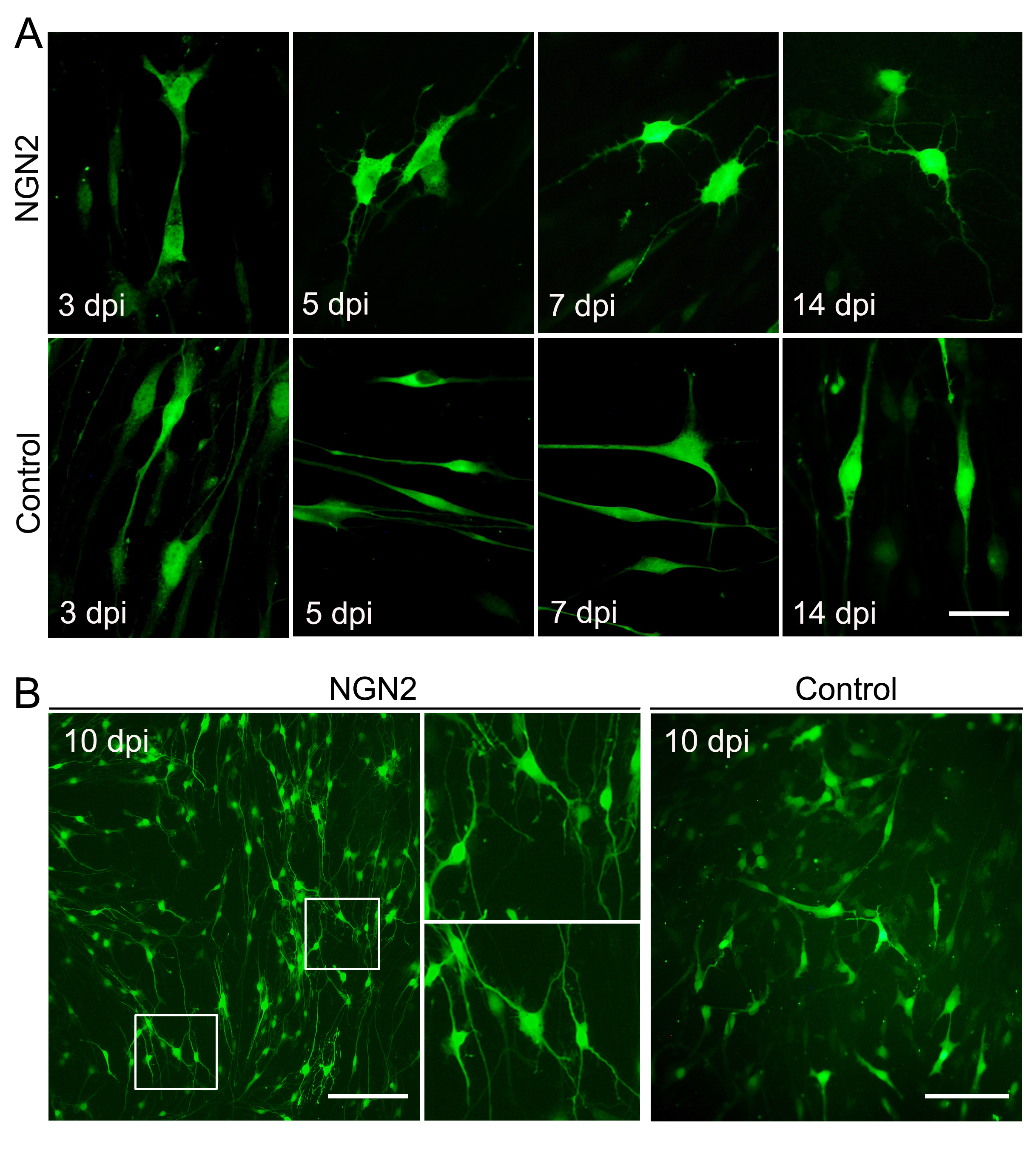


**Supplementary Figure 4 Cells changed from OEC morphology to neuronal morphology along the NGN2-mediated induction process.** (A) Time-course analysis of the rapid morphological changes of OECs induced by ectopic expression of NGN2. GFP-expressing virus was used as a control. (B) Representative images of cell morphology at 10 dpi. Higher magnification views of the boxed regions are also shown. Scale bar, 50 μm for (A); 100 μm for (B).


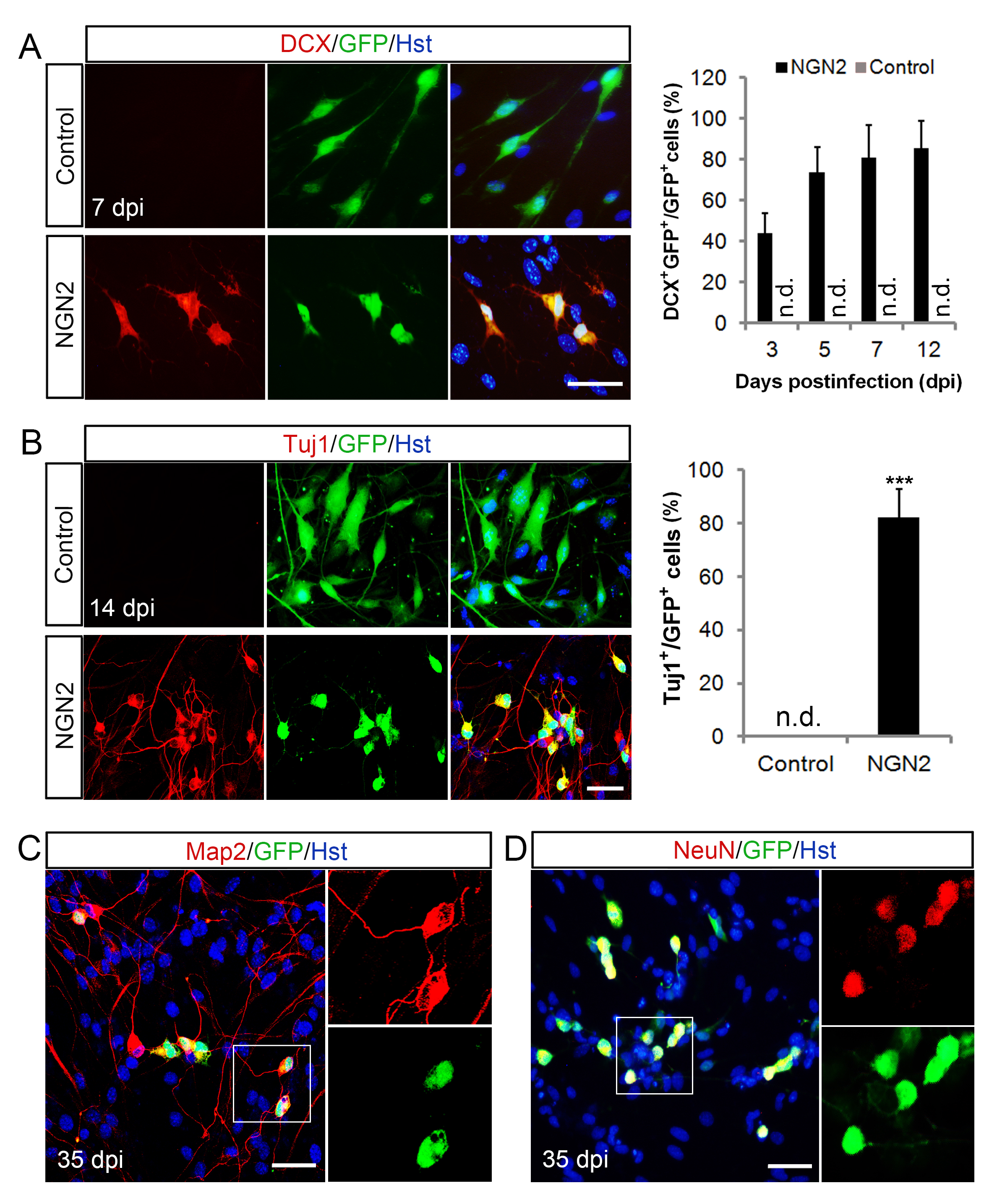


**Supplementary Figure 5** **Neuronal reprogramming of OECs by human GFAP promoter-driven NGN2. (A)** Quantitative analysis of NGN2-induced DCX^+^ cells during the indicated time course (n = 20 random fields from triplicate samples). **(B)** NGN2-induced neuronal reprogramming efficiency of adult olfactory bulb-derived OECs (n = 20 random fields from triplicate samples). **(C and D)** Immunocytochemical analysis of mature NGN2-induced neurons from adult olfactory bulb-derived OECs by staining with Map2 and NeuN at 35 dpi. n.d., not detected. ****P*<0.001 by Student’s *t* tests. Scale bar = 50 μm.


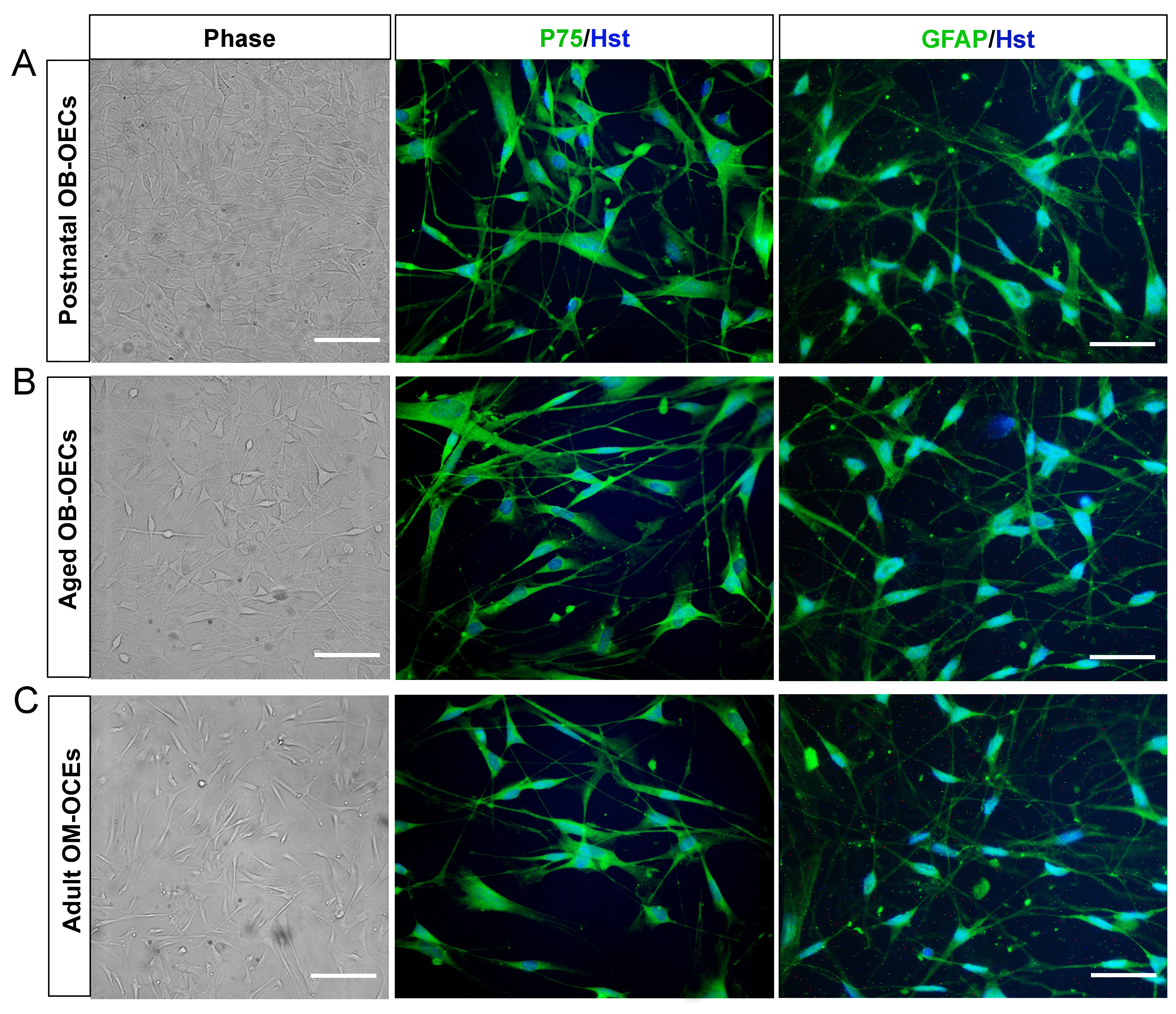


**Supplementary Figure 6 OEC culture of different origin.** OECs were cultured from postnatal olfactory bulb (Postnatal OB-OECs) (A), aged olfactory bulb (Aged OB-OECs) (B) and adult olfactory mucosa (Adult OM-OECs) (C). The identity of OECs was confirmed by staining with antibodies against P75 and GFAP. Scale bar, 100 μm for left panel; 50 μm for right panel.


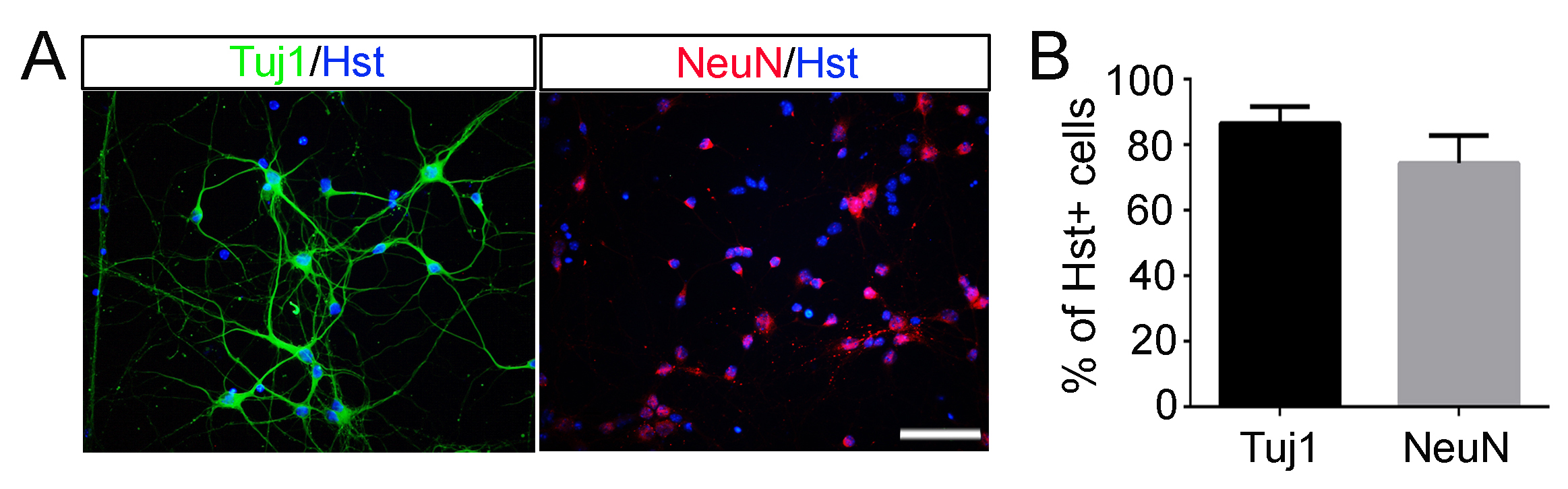


**Supplementary Figure 7 Cortical neurons cultured from postnatal mouse.** (A) The purity of cultured cortical neurons was determined by staining with antibodies against Tuj1 and NeuN. Nuclei were counterstained with Hst. (B) Quantification of the percentage of Tuj1^+^ or NeuN^+^ over Hst^+^ cells (n = 20 randomly selected fields from triplicate samples). Scale bar = 50 μm.


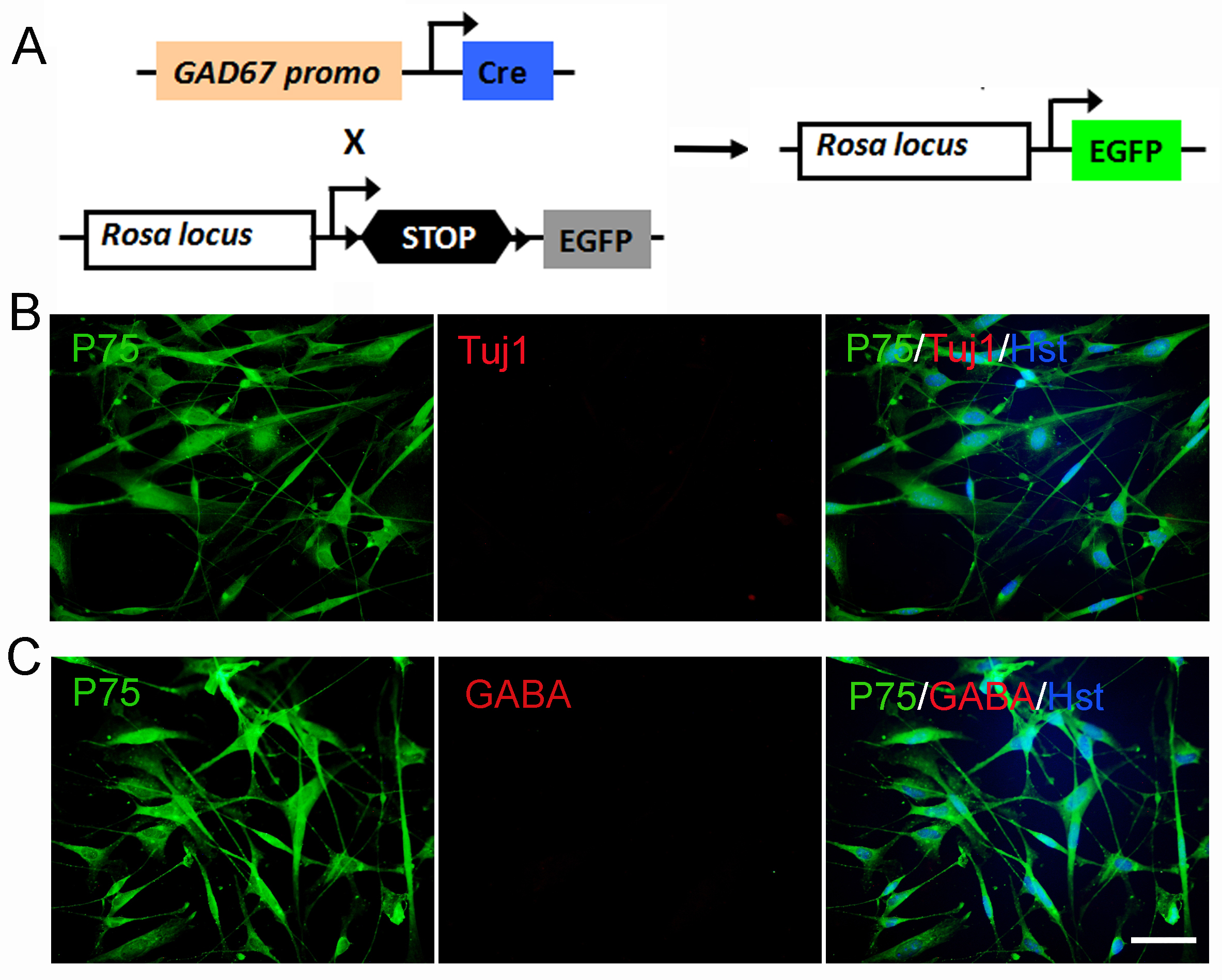


**Supplementary Figure 8 OECs cultured from GAD67-GFP transgenic mouse. (A)** A genetic reporting system for induced GABAergic neurons using GAD67-Cre;Rosa-EGFP mouse. **(B)** Immunocytochemical analysis of OECs cultured from GAD67-GFP transgenic mouse by staining with antibodies against P75, Tuj1 and GABA. Scale bar = 50 μm.

**Supplementary Table 1. Primary antibodies used for immunostaining**

**Antibody Host Dilution Source Catalog number**

Rat

Mouse

Rabbit

Rabbit

Mouse

Rabbit

Chicken

Rabbit

Rabbit

Mouse

Rabbit

Mouse

Mouse

RabbitMouse

Rabbit

Mouse

Mouse

Rabbit

1:200

1:100

1:100

1:100

1:100

1:100

1:600

1:100

1:100

1:100

1:100

1:200

1:100

1:100

1:100

1:500

1:500

1:100

1:100

Sigma

Sigma

Santa Cruz

Sigma

Sigma

Boster Biological Technology

AVES LABS

Wako

abcam

Millipore

Abcam

Millipore

Boster Biological Technology

Abcam

Boster

CST

Biolegend

Covance

Snaptic Systems

B5002

C8035

SC8066

A2052

G3893

BA0056

1020

019-19741

Ab32454

MAB377

ab109186

BM0104

MAB5554

Ab8874

BM0120

5297S

801202

MMS435P

135302

Brdu (5-bromodeoxyuridine)

CSPG (chondroitin sulfate proteoglycan )

DCX (Doublecortin)

GABA (γ-aminobutyric acid)

GFAP (glial fibrillary acidic protein)

GFP (green fluorescent protein)

IBA1 (ionized calcium binding adapter molecule 1)

Map2 (Microtubule-associated protein-2)

NeuN (neuron-specific nuclear protein)

Olig2 (oligodendrocyte transcription factor 2)

Pax6 (Paired box protein Pax-6)

PCNA (proliferating cell nuclear antigen)

P75 (p75 neurotrophin receptor)

S100β (Protein S100-B)

SYN-1 (Synapsin-1)

Tuj1 (Tubulin beta-3)

vGlut-1 (Vesicular glutamate transporter 1)
